# Adrenaline auto-injector devices: do current prescription and training measures work?

**DOI:** 10.1186/2045-7022-3-S3-P123

**Published:** 2013-07-25

**Authors:** S Alam, B Kasternow, J Lukawska, C Corrigan

**Affiliations:** 1 Allergy, Guy's and St Thomas' NHS Trust, London, UK

## Background

The incidence of systemic allergic reactions in the UK continues to rise. Appropriate and timely use of an adrenaline auto-injector is life saving. Anaphylaxis guidelines specify that individuals at high risk carry an adrenaline auto-injector at all times and receive adequate instruction on its use. We examined whether adult patients prescribed an adrenaline auto-injector had been given it appropriately and maintained and used it properly.

## Methods

Using a standard proforma we asked 100 new adult allergy clinic patients (38 male, mean age 33 yr) previously prescribed an adrenaline auto-injector in primary care, the ER or a paediatric allergy clinic whether they were currently carrying the device and whether it was in date. Patients were also asked to demonstrate its use to a trained observer.

## Results

84 patients had received the device for an appropriate indication, whereas in 16 it had been prescribed inappropriately by non-allergy centres. 80 patients had an in date device about their person. 55 failed at various steps to use their device correctly. Of the 45 who did use it correctly, 15 had been trained at paediatric allergy centres more than once, 9 had received a single demonstration in primary care (4) or the ER (5), and 21 could not remember where their device was issued. All patients failing demonstration of correct usage had received a single training session.

## Conclusion

A significant proportion of patients in the UK are prescribed adrenaline auto-injectors inappropriately at non-specialist centres. Our data are consistent with the hypothesis that repeated training maintains adequate technique. We suggest that technique should be reviewed at every opportunity in the allergy clinic and regularly in primary care.

## Disclosure of interest

None declared.

